# Supplementary material for: Selecting High-Dimensional Representations of Physical Systems by Reweighted Diffusion Maps
Source: J Phys Chem Lett. 2023 Mar 10;14(11):2778–83. doi: 10.1021/acs.jpclett.3c00265 (PMC10041639; doi:10.1021/acs.jpclett.3c00265)
Supplement: Supplementary file 1 — jz3c00265_si_001.pdf [file jz3c00265_si_001.pdf]

Supporting Information:

Selecting High-Dimensional Representations of Physical Systems by Reweighted Diffusion Maps

Jakub Rydzewski*

Institute of Physics, Faculty of Physics, Astronomy and Informatics, Nicolaus Copernicus University, Grudziadzka 5, 87-100 Toruń, Poland

E-mail: jr@fizyka.umk.pl

Simulation Data Sets

- m-Dimensional hypercube: The data sets for $m = 4, 6, 8, 10$ informative variables of 2500 samples are generated using `scikit learns`'s function `make_classification`.[S1]

- Alanine dipeptide: The GROMACS 2019.2 code[S2] patched with a development version of the PLUMED plugin[S3,S4] are used to perform a 100-ns alanine dipeptide (Ace-Ala-Nme) simulation. The Amber99-SB force field is used,[S5] and a time step of 2 fs. The simulation is carried out in the canonical ensemble at 300 K using the stochastic velocity rescaling thermostat[S6] with a relaxation time of 0.1 ps. Hydrogen bonds are constrained using LINCS.[S7] The simulations are performed in vacuum without periodic boundary conditions and with no cut-offs for electrostatic and non-bonded van der Waals interactions. Well-tempered metadynamics[S8] with a bias factor of 5 is used to enhance the fluctuations of the backbone dihedral angles Φ and Ψ of alanine dipeptide. An initial Gaussian height is set to 1.2 kJ/mol, a Gaussian width to 0.2 rad, and Gaussians are deposited every 1 ps. The time-dependent constant $c(t)$ required

to perform the Tiwary–Parrinello reweighting[S9] is updated every time a Gaussian is added. To generate a biased data set, the last 10 ns of the run sampled every 4 ps (2500 samples in total) is used.

- Chignolin: A 1-μs chignolin simulation starting from an NMR structure[S10] is performed using the GROMACS 2019.2 code[S2] patched with a development version of the PLUMED plugin.[S3,S4] The CHARMM27 force field[S11] is used. The simulation is carried out with a time step of 2 fs in the canonical ensemble using the stochastic velocity rescaling thermostat[S6] at 340 K with a relaxation time of 0.1 ps. Hydrogen bonds are constrained using LINCS.[S7] The simulation is performed in solution (TIP3P) with periodic boundary conditions and cut-offs for electrostatic and non-bonded van der Waals interactions of 1 nm. Well-tempered metadynamics[S8] with of bias factor of 5 is used to enhance the fluctuations of the distance between the Cα atoms of Y1 and Y10 and the radius of gyration. Gaussian are deposited every 1 ps with an initial Gaussian height of 2 kJ/mol and Gaussian widths of 0.1 nm. The time-dependent constant $c(t)$ required to perform the Tiwary–Parrinello reweighting[S9] is estimated every 50 added Gaussians. To generate a biased data set, the last 20 ns of the run sampled every 8 ps (2500 samples in total) is used.

Constructing Markov Transition Matrices

- Scale constant ε: The median of pairwise Euclidean distances between samples is used to estimate ε. Before calculating the median, we sort the pairwise distances and skip around 1% of them to make ε less sensitive to outliers.

- The number of eigenvalues in the spectral loss σ_d: As the eigenvalues of the Markov transition matrix decay exponentially, it is important to include the dominant eigenvalues for calculating the spectral loss. Depending on the effective timescales observed in the system, the decay can be slow (this can vary especially for not optimal partial

high-dimensional representations). Therefore we consider 50 dominant eigenvalues to estimate σ_d.

- Sequential selection algorithm: Instead of performing a brute-force search through every combination of configuration variables, we use an algorithm that provides a sub-optimal solution known as sequential backward floating selection[S12] (see the main text). The search method is trivially parallelizable. Each minimization of the spectral loss for the high-dimensional systems considered in the main text takes ∼100 seconds on 32 CPU cores.

Selected Configuration Variables

Table S1: Selected configuration variables (distances between atom k and atom l) of alanine dipeptide for $d = 10$.

Number	Atom k	Atom l	Selection
1	Ace CH3	Ace C	
2	Ace CH3	Ace O	
3	Ace CH3	Ala N	
4	Ace CH3	Ala CA	
5	Ace CH3	Ala CB	yes
6	Ace CH3	Ala C	yes
7	Ace CH3	Ala O	
8	Ace CH3	Nme N	
9	Ace CH3	Nme CH3	
10	Ace C	Ace O	
11	Ace C	Ala N	
12	Ace C	Ala CA	
13	Ace C	Ala CB	
14	Ace C	Ala C	yes
15	Ace C	Ala O	
16	Ace C	Nme N	
17	Ace C	Nme CH3	
18	Ace O	Ala N	
19	Ace O	Ala CA	yes
20	Ace O	Ala CB	
21	Ace O	Ala C	
22	Ace O	Ala O	
23	Ace O	Nme N	
24	Ace O	Nme CH3	yes
25	Ala N	Ala CA	
26	Ala N	Ala CB	
27	Ala N	Ala C	
28	Ala N	Ala O	
29	Ala N	Nme N	yes
30	Ala N	Nme CH3	yes
31	Ala CA	Ala CB	
32	Ala CA	Ala C	
33	Ala CA	Ala O	
34	Ala CA	Nme N	
35	Ala CA	Nme CH3	
36	Ala CB	Ala C	
37	Ala CB	Ala O	yes
38	Ala CB	Nme N	
39	Ala CB	Nme CH3	yes
40	Ala C	Ala O	
41	Ala C	Nme N	
42	Ala C	Nme CH3	
43	Ala O	Nme N	
44	Ala O	Nme CH3	yes
45	Nme N	Nme CH3	

Table S2: Selected configuration variables (dihedral angles) of chignolin for $d = 16$.

Number	Variable	Residues	Selection
1	$\sin\Psi_1$	Y1–Y2	yes
2	$\cos\Psi_1$	Y1–Y2	yes
3	$\sin\Phi_2$	Y1–Y2	
4	$\cos\Phi_2$	Y1–Y2	yes
5	$\sin\Psi_2$	Y2–D3	yes
6	$\cos\Psi_2$	Y2–D3	yes
7	$\sin\Phi_3$	Y2–D3	
8	$\cos\Phi_3$	Y2–D3	yes
9	$\sin\Psi_3$	D3–P4	
10	$\cos\Psi_3$	D3–P4	
11	$\sin\Phi_4$	D3–P4	yes
12	$\cos\Phi_4$	D3–P4	
13	$\sin\Psi_4$	P4–E5	yes
14	$\cos\Psi_4$	P4–E5	
15	$\sin\Phi_5$	P4–E5	
16	$\cos\Phi_5$	P4–E5	
17	$\sin\Psi_5$	E5–T6	yes
18	$\cos\Psi_5$	E5–T6	
19	$\sin\Phi_6$	E5–T6	
20	$\cos\Phi_6$	E5–T6	yes
21	$\sin\Psi_6$	T6–G7	
22	$\cos\Psi_6$	T6–G7	
23	$\sin\Phi_7$	T6–G7	yes
24	$\cos\Phi_7$	T6–G7	
25	$\sin\Psi_7$	G7–T8	yes
26	$\cos\Psi_7$	G7–T8	
27	$\sin\Phi_8$	G7–T8	
28	$\cos\Phi_8$	G7–T8	yes
29	$\sin\Psi_8$	T8–W9	
30	$\cos\Psi_8$	T8–W9	
31	$\sin\Phi_9$	T8–W9	
32	$\cos\Phi_9$	T8–W9	yes
33	$\sin\Psi_9$	W9–Y10	yes
34	$\cos\Psi_9$	W9–Y10	
35	$\sin\Phi_{10}$	W9–Y10	
36	$\cos\Phi_{10}$	W9–Y10	yes

References

(S1) Pedregosa, F.; Varoquaux, G.; Gramfort, A.; Michel, V.; Thirion, B.; Grisel, O.; Blondel, M.; Prettenhofer, P.; Weiss, R.; Dubourg, V.; Vanderplas, J.; Passos, A.; Cournapeau, D.; Brucher, M.; Perrot, M.; Duchesnay, E. Scikit-learn: Machine Learning in Python. *J. Mach. Learn. Res.* **2011**, *12*, 2825–2830.

(S2) Abraham, M. J.; Murtola, T.; Schulz, R.; Páll, S.; Smith, J. C.; Hess, B.; Lindahl, E. GROMACS: High Performance Molecular Simulations through Multi-Level Parallelism from Laptops to Supercomputers. *SoftwareX* **2015**, *1–2*, 19–25.

(S3) Tribello, G. A.; Bonomi, M.; Branduardi, D.; Camilloni, C.; Bussi, G. PLUMED 2: New Feathers for an Old Bird. *Comp. Phys. Commun.* **2014**, *185*, 604–613.

(S4) PLUMED Consortium, Promoting Transparency and Reproducibility in Enhanced Molecular Simulations. *Nat. Methods* **2019**, *16*, 670–673.

(S5) Hornak, V.; Abel, R.; Okur, A.; Strockbine, B.; Roitberg, A.; Simmerling, C. Comparison of Multiple Amber Force Fields and Development of Improved Protein Backbone Parameters. *Proteins* **2006**, *65*, 712–725.

(S6) Bussi, G.; Donadio, D.; Parrinello, M. Canonical Sampling through Velocity Rescaling. *J. Chem. Phys.* **2007**, *126*, 014101.

(S7) Hess, B. P-LINCS: A Parallel Linear Constraint Solver for Molecular Simulation. *J. Chem. Theory Comput.* **2008**, *4*, 116–122.

(S8) Barducci, A.; Bussi, G.; Parrinello, M. Well-Tempered Metadynamics: A Smoothly Converging and Tunable Free-Energy Method. *Phys. Rev. Lett.* **2008**, *100*, 020603.

(S9) Tiwary, P.; Parrinello, M. A Time-Independent Free Energy Estimator for Metadynamics. *J. Phys. Chem. B* **2015**, *119*, 736–742.

(S10) Honda, S.; Akiba, T.; Kato, Y. S.; Sawada, Y.; Sekijima, M.; Ishimura, M.; Ooishi, A.; Watanabe, H.; Odahara, T.; Harata, K. Crystal Structure of a Ten-Amino Acid Protein. *J. Am. Chem. Soc.* **2008**, *130*, 15327–15331.

(S11) Mackerell Jr, A. D.; Feig, M.; Brooks III, C. L. Extending the Treatment of Backbone Energetics in Protein Force Fields: Limitations of Gas-Phase Quantum Mechanics in Reproducing Protein Conformational Distributions in Molecular Dynamics Simulations. *J. Comput. Chem.* **2004**, *25*, 1400–1415.

(S12) Pudil, P.; Novovičová, J.; Kittler, J. Floating Search Methods in Feature Selection. *Pattern Recognit. Lett.* **1994**, *15*, 1119–1125.
